# Biomechanical effects of S1 sacroiliac screws versus S2 sacroiliac screws on sacroiliac screws combined with a lumbar iliac fixation in the treatment of vertical sacral fractures: a biomechanical finite element analysis

**DOI:** 10.1186/s12891-023-06884-y

**Published:** 2023-09-22

**Authors:** Yupeng Ma, Tao Huang, Weiwei Liu, Huanyu Hong, Yong Zhao, Jiangtao Lin, Yu Li

**Affiliations:** 1 First Ward of Trauma Orthopaedics, Yantai Shan Hospital, Yantai, Shandong Province 264003 P.R. China; 2 Yantai Key Laboratory for Repair and Reconstruction of Bone & Joint, Yantai, Shandong Province 264003 P.R. China; 3 Orthopaedics Department, Yantai Shan Hospital, Yantai, Shandong Province 264003 P.R. China

**Keywords:** Sacral fractures, Sacroiliac screws, Lumbar-iliac fixation, Biomechanics

## Abstract

**Objective:**

To examine the impact of sacroiliac screw position and length on the biomechanical properties of triangular osteosynthesis in treating unilateral vertical sacral fractures and provide a clinical reference.

**Methods:**

Unilateral Denis type II sacral fractures were modelled using finite elements to represent Tile C pelvic ring injuries. Six sacroiliac screws were used with iliolumbar fixation patterns to fix the sacral fractures, and the sacral stability, maximum pressure, and stress distribution were compared among the internal fixation modalities.

**Results:**

The best vertical stability of the internal fixation model was achieved when the S1 segment was fixed with lengthened sacroiliac screws, followed by when the S1 segment was fixed using normal sacroiliac screws. There was no significant difference in vertical stability between the S1 + S2 dual-segment fixation model and the S1-segment fixation model. The maximum pressure under a vertical force of 600 N showed a trend of L5LS1 < L5NS1 < L5LS12 < L5LS2 < L5NS2 < L5NS12.

**Conclusions:**

In unilateral vertical sacral fractures (Denis II) treated with triangular osteosynthesis using triangular jointing combined with unilateral iliolumbar + sacroiliac screw fixation, the use of a single lengthened sacroiliac screw for the S1 segment is recommended to achieve the best vertical stability of the sacrum with less maximum compression on the internal fixation components. If it is not possible to apply a lengthened sacroiliac screw, the use of a normal sacroiliac screw for the S1 segment is recommended. Adding an S2 screw does not significantly increase the vertical stability of the sacrum.

## Introduction

The sacrum is a vital structure that forms the posterior pelvic ring and distributes gravitational force from the spine to the acetabulum on both sides. High-energy injuries mainly cause unstable vertical sacral fractures, often combined with nerve injuries and multiple traumas resulting in instability of the pelvic ring. Unstable sacral fractures are associated with high mortality and morbidity if not treated properly [1–3].

Open or closed reduction with internal fixation has become the gold standard for treating these unstable fractures because of the advancements in procedures and suitable fixation devices. Surgery for a fracture should restore anatomical integrity, release pressure on nerves, and provide sufficient stability for immediate activity.

To restore the posterior pelvic ring, internal fixation techniques using components such as sacral rods [4], transiliac rods [5–7], percutaneous iliosacral screws [8, 9], and posterior microplates [10] have been employed. However, none of these fixation systems can allow unrestricted weight-bearing, resist shear vertical pressures, or enable safe mobility following surgery.

Triangular osteosynthesis is a composite fixation method consisting of a longitudinal fixation system combined with a transverse fixation system. The combined use of common lumbar and pelvic instrumentation and iliosacral screws or transiliac plates for horizontal fixation was first described by Shildhauer et al. [11]. They showed that triangular osteosynthesis provides better fixation strength and enables early weight-bearing [12, 13].

Sacroiliac screws are widely used in the minimally invasive treatment of sacral fractures. The safety and effectiveness of sacroiliac screws have significantly increased with the development of navigation and positioning techniques. In triangular osteosynthesis systems, sacroiliac screws are commonly used for lateral stabilization. However, according to the literature, the effect of increasing the length or changing the position of the sacroiliac screw on the biomechanical properties of triangular osteosynthesis remain unclear. We hypothesized that increasing the length of sacroiliac screws would increase the stability of sacral fracture fixation. Sacroiliac screws in S1 segments in conjunction with S2 segments increased the stability of sacral fractures after fixation.Therefore, in this study, the effect of changing the position and length of the sacroiliac screw on the biomechanical characteristics of triangular osteosynthesis was investigated by a three-dimensional finite element method to provide a theoretical basis for internal fixation device selection.

## Methods

This study was approved by the ethics committee of Yantai Shan Hospital and was carried out in accordance with the ethical standards of the Declaration of Helsinki. Informed consent was obtained from each participant included in the study.

### Construction of a finite element model

**Fig. 1:**
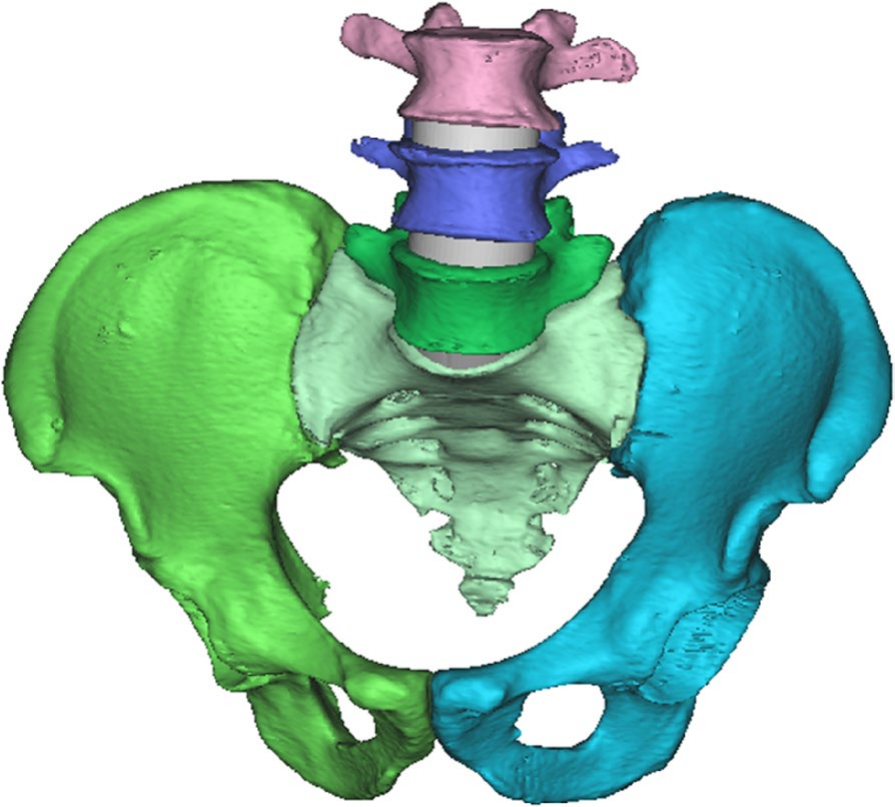
Individual pelvic components generated from CT data

This study used data from a 64-slice spiral CT (Philips) scan of the pelvis and L3-L5 segment of a healthy adult female (165 cm, 35 years, 65 kg). The slice thickness was 1 mm. Using image processing software (Mimics 17.0,Materialise, Belgium), we produced a virtual 3D model of the lumbar spine and pelvis from CT data in Digital Imaging and Communications in Medicine (DICOM) format. The CT grey value-based segmentation technique was applied to generate the individual components, as shown in Fig. 1. The 3D model of the pelvis obtained in Mimics 17.0 medical image processing software (Materialise, Belgium) was imported into 3-matic software (Materialise, Belgium) for model smoothing to enable further model operations.

**Fig. 2:**
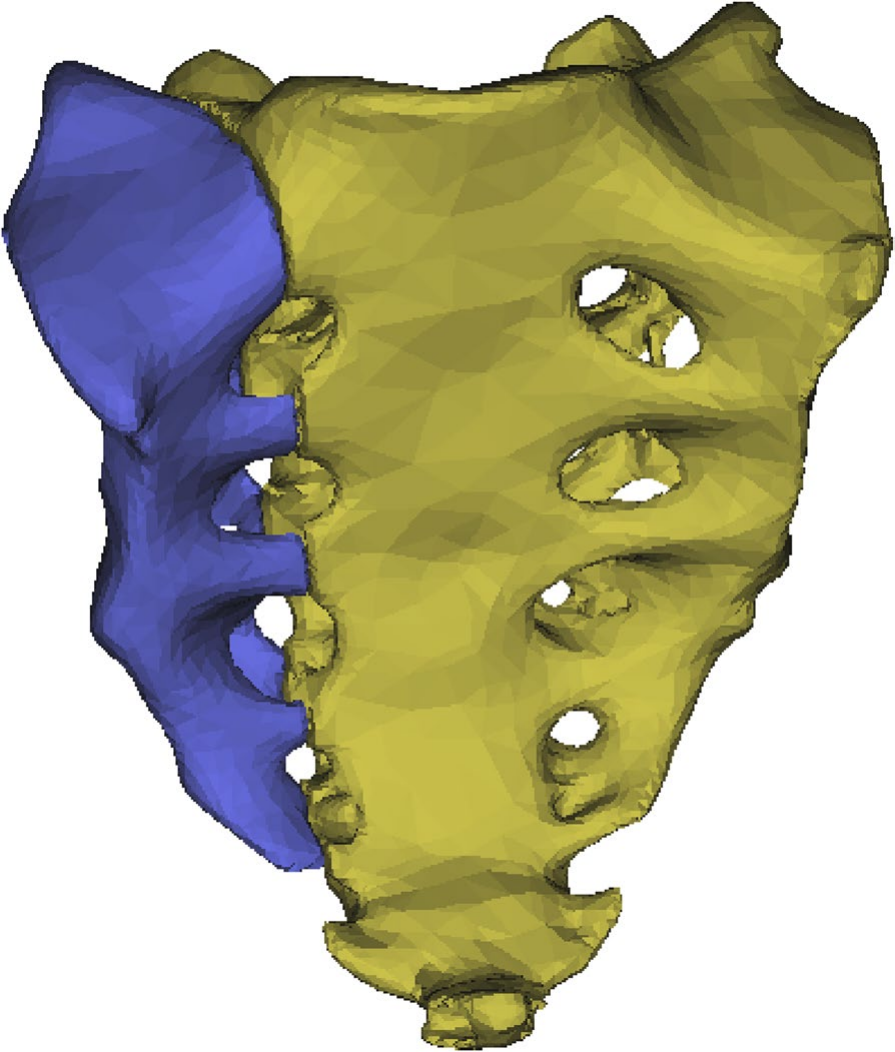
Unilateral longitudinal fracture of the sacrum through the sacral foramen (AO C1.3, Denis II)

To create a sacral fracture model (AO C1.3, Denis II), we split the original sacral model longitudinally along the sacral foramen into two parts,as shown in Fig. 2. We simulate an AO C1.3 pelvic ring injury in the Tile-AO-Müller subtype of pelvic fractures. In this case, the posterior ring injury is a longitudinal fracture of the sacrum through the sacral foramen. We assume that the anterior ring injury returns to normal stability with fixation. This excludes the effect of different fixation methods for anterior ring injuries on the outcome of the posterior ring. The model components were meshed using the Remesh module in 3-matic. The mesh consisted of tetrahedra with four nodes and three degrees of freedom. To assign material parameters, the mesh model was transferred back to Mimics. The material qualities were set as nonhomogeneous and isotropic.

Different skeletal components of the model were determined using a greyscale-based technique and a formula in Mimics that divides the greyscale values into ten levels. The formula for material assignments was based on previous reports in the literature [14].

SolidWorks 2017 software (Dassault Systemes S.A., USA) was used to model the implants. Mesh creation was performed after the model was imported into 3-matic software for pelvic bone model assembly. The implant was made of titanium alloy, which was imported into Mimics for material property assignment.

### Establishment of ligament and muscle models and application of loads

Abaqus 6.13 software (Simulia, Providence, RI, USA) was used to import the mesh models of the bones and screws, and spring-damping cells were then utilized to simulate the ligaments and muscles. Tables 1 and 2 display the model material parameter settings and the ligament parameter settings [15–18].

**Table 1:** Lumbar spine material parameters

Material	Elastic modulus, MPa	Poisson ratio	Cross-sectional area, mm2
Disc annulus	8.4	0.45	
Disc nucleus	Mooney–Rivlin c1 = 0.12, c2 = 0.03		
Anterior longitudinal ligament	7		63.7
Posterior longitudinal ligament	7		20
Ligamentum flavum	3		40
Intertransverse ligament	7		1.8
Capsular ligament	4		30
Interspinous ligament	6		40
Supraspinous ligament	6.6		30
Implants	11,400	0.3

**Table 2:** Pelvic ligament parameters

Material	K, N/m	Number of springs
Anterior and capsule sacroiliac ligament	700	27
Posterior sacroiliac ligament	1400	15
Interosseous sacroiliac ligament	2800	8
Iliolumbar ligament	2800	30
Sacrospinous ligament	1400	9
Sacrotuberous ligament	1500	15
Superior pubic ligament	500	24
Arcuate pubic ligament	500	24

The pubic symphysis and the sacroiliac joint were established as bound limitations. At the bilateral acetabular nodes, six degrees of freedom were constrained. To simulate the effect of gravity on the human body when standing up straight, a force of 600 N was applied vertically downwards on the surface of the upper endplate of the L3 vertebra.

**Fig. 3:**
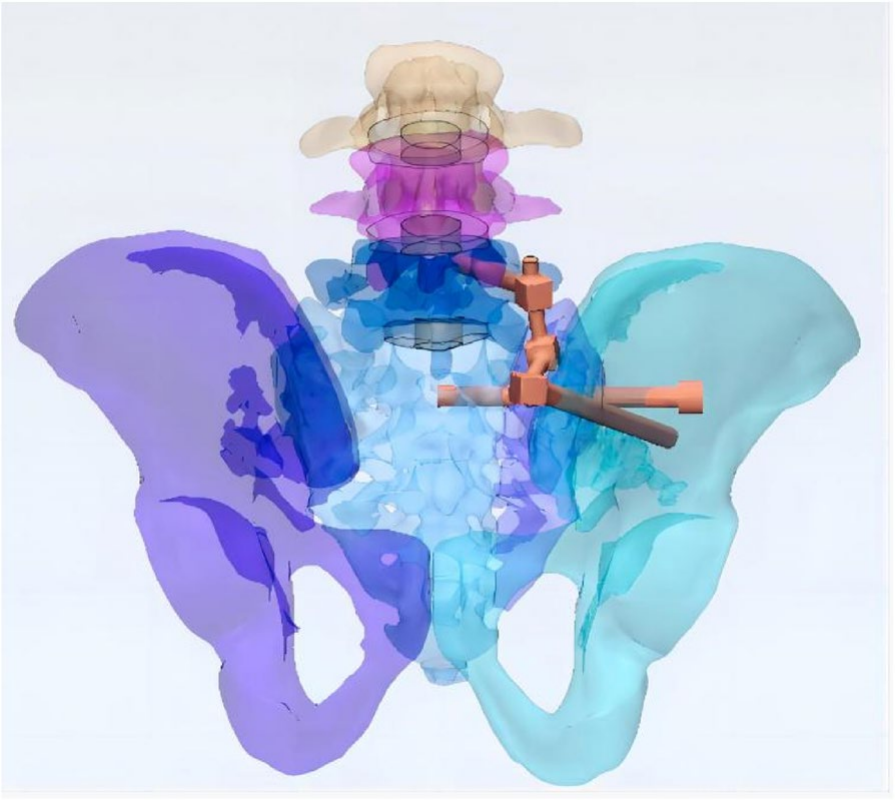
L5NS1 fixation model

**Fig. 4:**
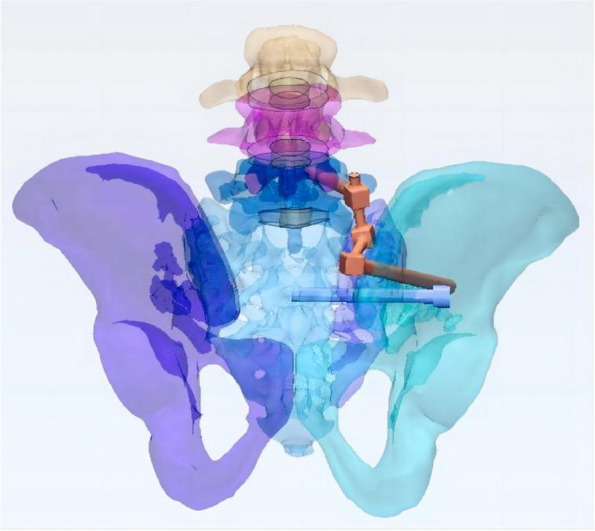
L5NS2 fixation model

**Fig. 5:**
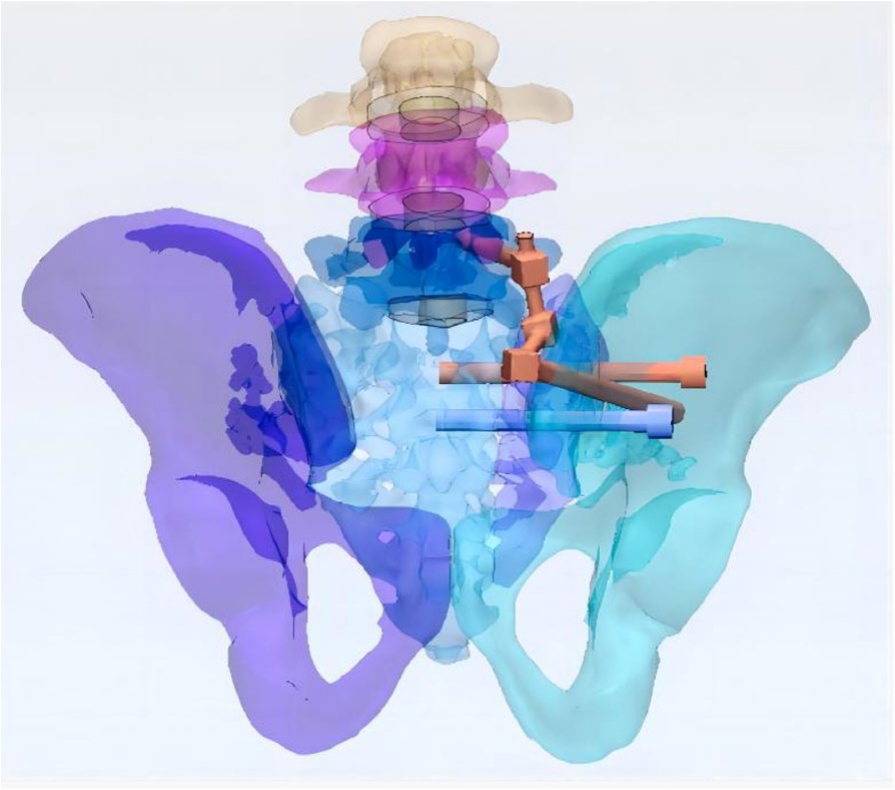
L5NS12 fixation model

**Fig. 6:**
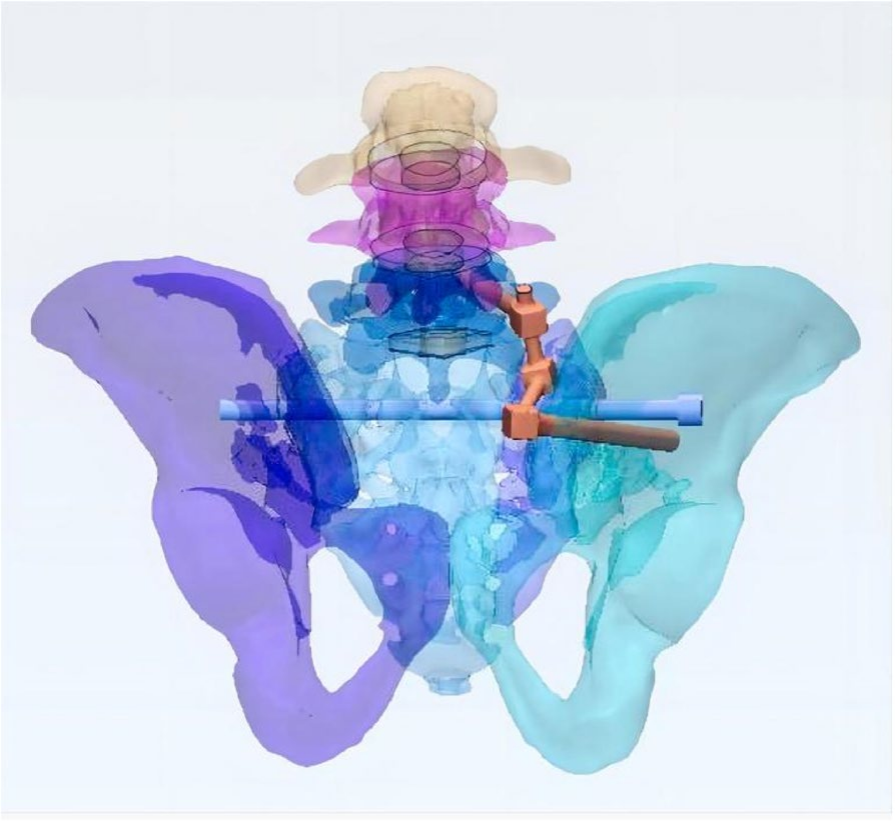
L5LS1 fixation model

**Fig. 7:**
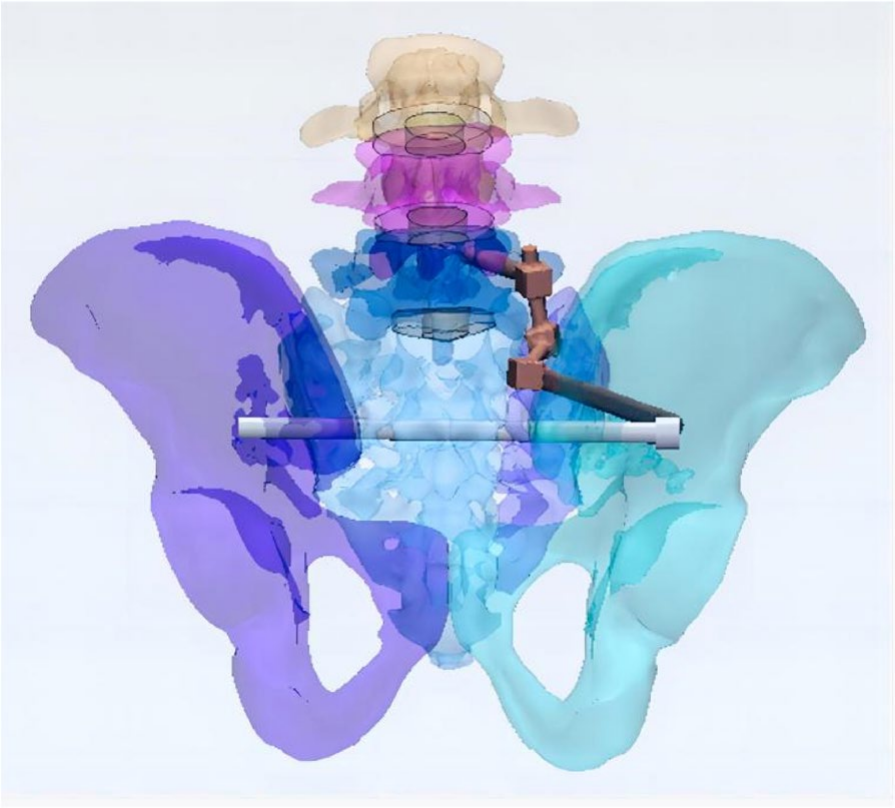
L5LS2 fixation model

**Fig. 8:**
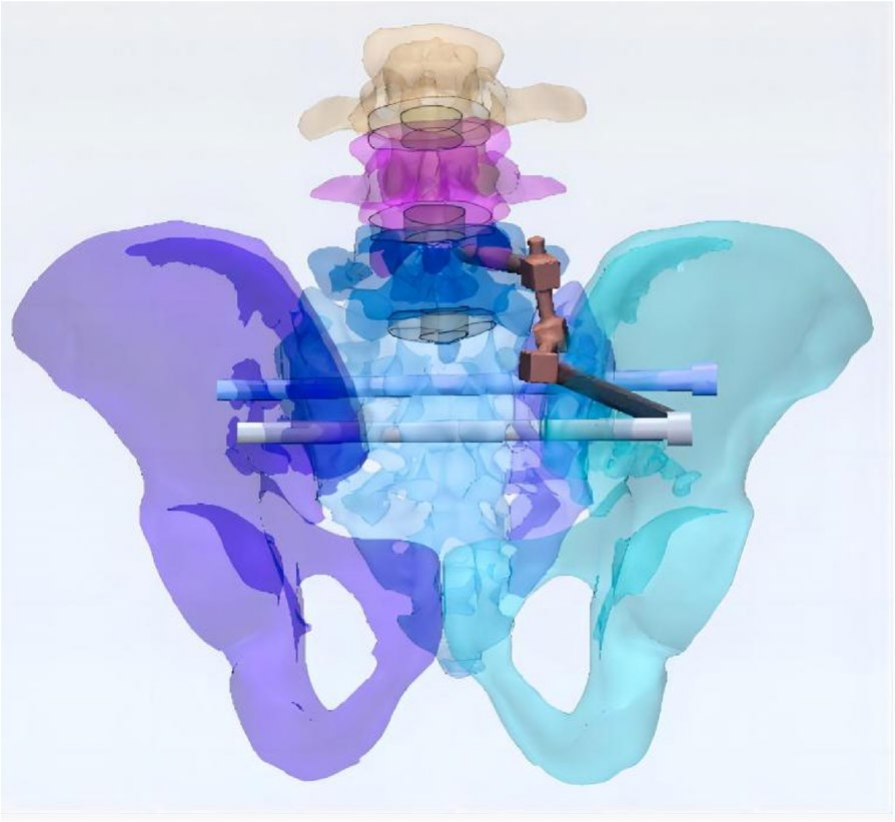
L5LS12 fixation model

In this study, a normal sacroiliac screw was one whose length extended past the fracture line to the midline of the sacrum. Sacroiliac screws that were lengthened were those that passed the fracture line and entered the opposing iliac bone. In this investigation, six internal fixation models were developed, as follows (Figs. 3, 4, 5, 6, 7 and 8): (1) unilateral L5 iliolumbar + S1 normal sacroiliac screw fixation (L5NS1); (2) unilateral L5 iliolumbar + S2 normal sacroiliac screw fixation (L5NS2); (3) unilateral L5 iliolumbar + S1S2 normal sacroiliac screw fixation (L5NS12); (4) unilateral L5 iliolumbar + S1 lengthened sacroiliac screw fixation (L5LS1); (5) unilateral L5 iliolumbar + S2 lengthened sacroiliac screw fixation (L5LS2); and (6) unilateral L5 iliolumbar + S1S2 lengthened sacroiliac screw fixation (L5LS12). The lumbar pedicle screws and iliac screws had a length and diameter of 45 mm and 6.5 mm and 70 mm and 7.5 mm, respectively. The sacroiliac screws had a diameter of 7.3 mm. The titanium alloy material parameters were assigned.

### Finite element model validation

**Fig. 9:**
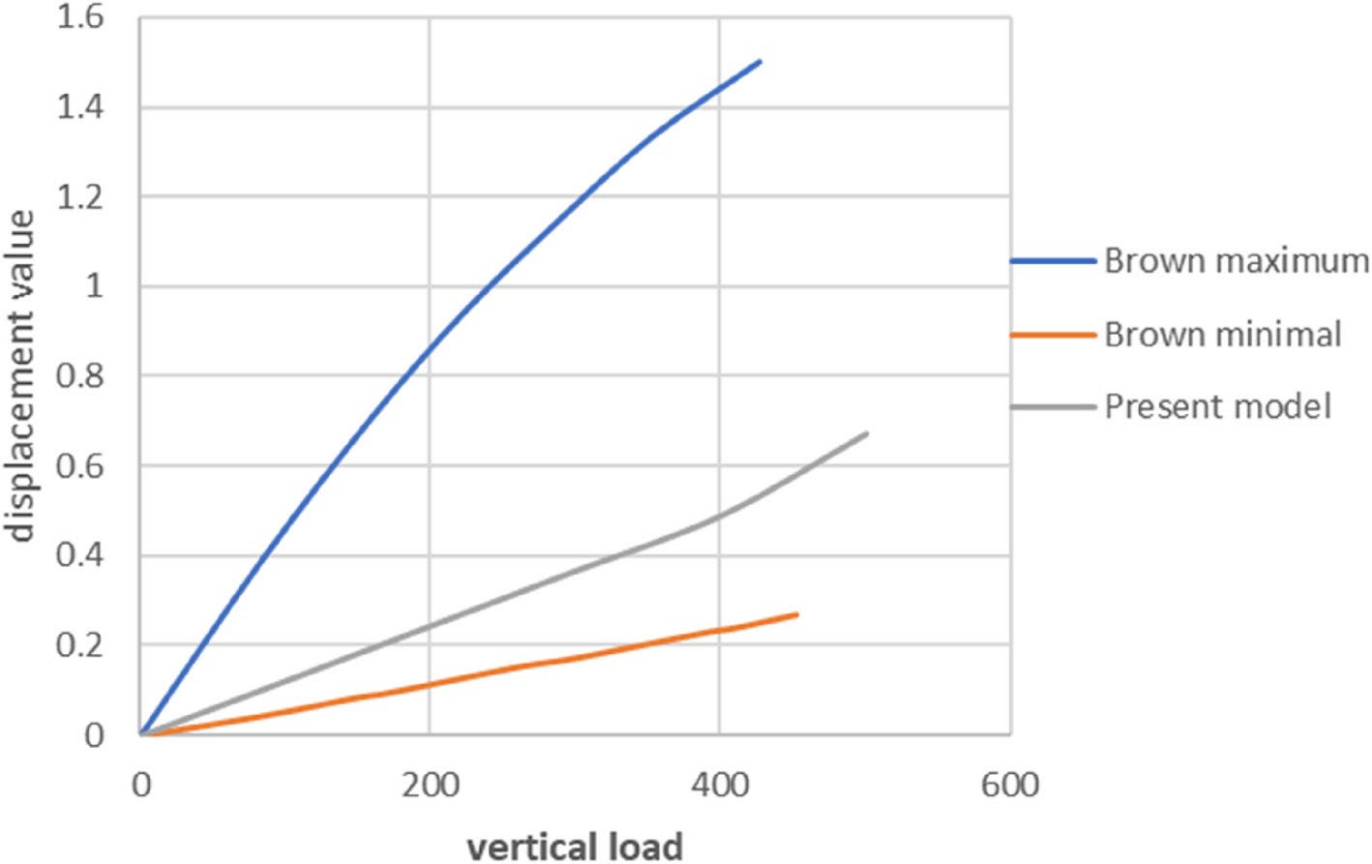
Simulation of Brown’s in vitro mechanical experiment to obtain displacement curves for the model

In this study, the spinal and pelvic finite element models were validated. The vertical displacement experiment in the spinal model validation simulated Brown's in vitro experiment [19], as shown in Fig. 9. The data of the L4 vertebra under forwards flexion, backwards extension, lateral bending, and torsional movements were compared with the in vitro data reported by Markolf; the data from both groups were similar, as were the deformation trends [20]. Data from the pelvic model were compared with data from the Miller model. The model was tested using five translational loads (294 N) and three rotational load moments (42 N m) (anterior, posterior, superior, inferior, mediolateral, flexion, extension, and axial rotation). The recorded results were compared with those of the Miller model. The various test data of this model were within the standard errors of the Miller model data and generally in good agreement [21].

### Experimental measurements

A vertical force of 600 N was applied to the upper surface of the sacrum to simulate the pressure on the sacrum in a standing situation. Boolean operations were applied to the six internal fixation models. The normal model was compared with the six internal fixation models mentioned above in terms of vertical displacement. To assess the stress distribution of the internal fixation components, the maximum pressure on the fixation components was measured, and the distribution of stress on the fixation configuration was examined.

## Results

### Vertical sacral displacement

Under 600 N of vertical pressure, the vertical displacement of five selected points (A-E) on the upper surface of the sacrum was recorded for the six models. The results are shown in Table 3.

**Table 3:** Vertical displacement of the upper surface of the sacrum at points A-E

Model	A(mm)	B(mm)	C(mm)	D(mm)	E(mm)	Mean(SD)
L5NS1	0.397	0.3168	0.3611	0.4375	0.3677	0.376 ± 0.0448
L5NS2	0.5399	0.4137	0.4284	0.5649	0.4816	0.4857 ± 0.0665
L5LS1	0.3218	0.2527	0.2845	0.3521	0.2937	0.301 ± 0.0378
L5LS2	0.5235	0.4159	0.4445	0.5515	0.4709	0.4813 ± 0.0558
L5NS12	0.4209	0.3302	0.3605	0.447	0.3761	0.3869 ± 0.0469
L5LS12	0.29	0.2121	0.2303	0.3023	0.2683	0.2606 ± 0.0385

**Fig. 10:**
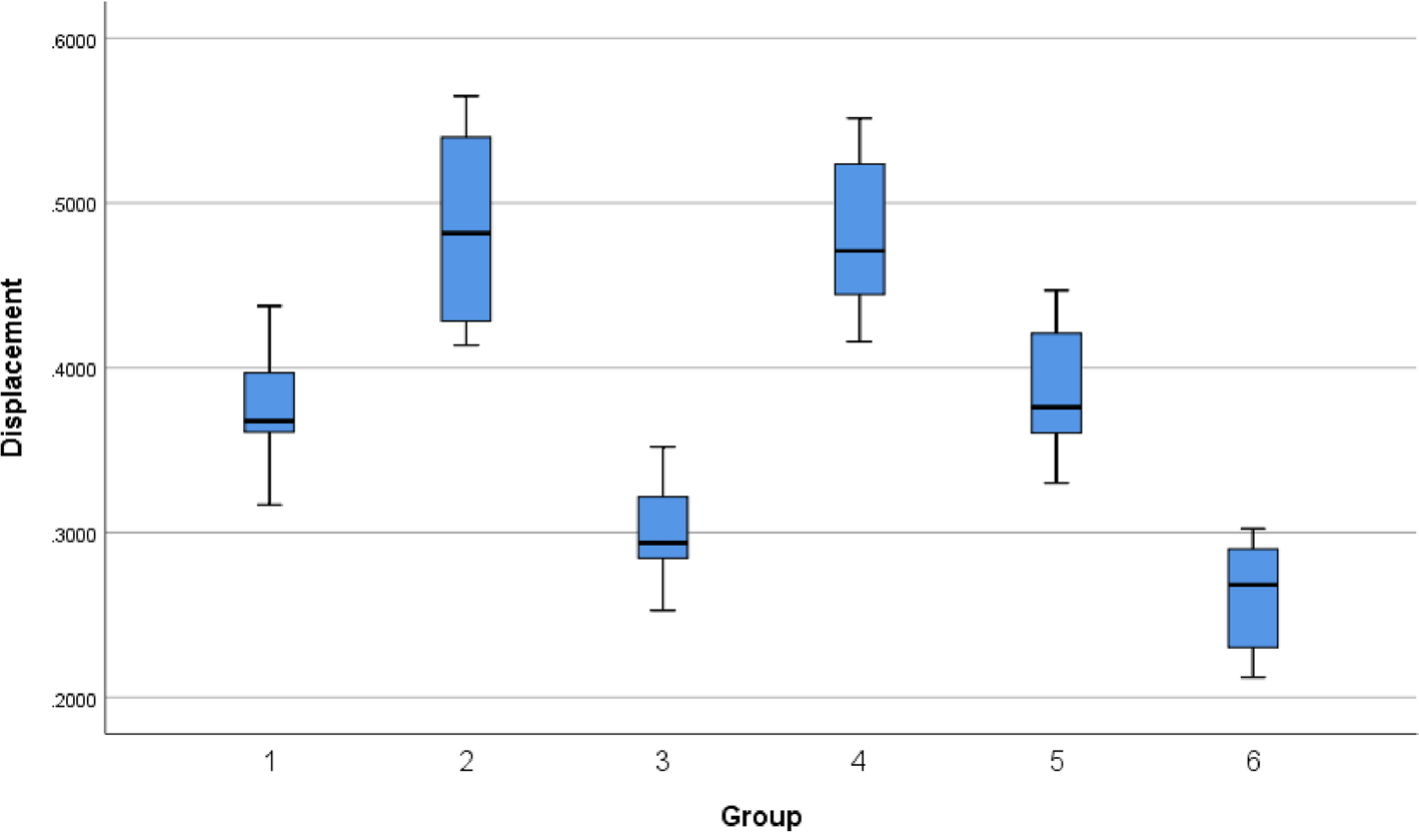
Displacement of the upper surface of the sacrum in the standing position

The vertical sacral displacement followed a trend of L5LS12 < L5LS1 < L5NS1 < L5NS12 < L5LS2 < L5NS2; the values are shown in Table 3 and Fig. 10. To identify statistically significant differences in vertical displacement among the models, statistical analyses were performed using SPSS 26.0 statistics software (IBM, USA). A normality test was performed on the vertical sacral displacement data to verify whether the data conformed to a normal distribution. The Kolmogorov–Smirnov (V) test and the Shapiro‒Wilk test were also performed on the vertical sacral displacement data, and the results showed *P* > 0.05 for both normality tests for all six groups, indicating that the data in all six groups conformed to a normal distribution (Table 4). The vertical displacement data from the six groups were subjected to the chi-square test, which showed *p* > 0.05 (Table 5).

**Table 4:** Test of normality

Tests of Normality							
	Group	Kolmogorov‒Smirnov^a^			Shapiro‒Wilk		
		Statistic	df	Sig	Statistic	df	Sig
Displacement	1	.174	5	.200^b^	.985	5	.961
	2	.206	5	.200^b^	.917	5	.513
	3	.176	5	.200^b^	.988	5	.973
	4	.176	5	.200^b^	.958	5	.793
	5	.191	5	.200^b^	.967	5	.853
	6	.184	5	.200^b^	.932	5	.610

Group 1: L5NS1; Group 2: L5NS2; Group 3: L5LS1; Group 4: L5LS2; Group 5: L5NS12; Group 6: L5LS12

^a^Lilliefors significance correction

^b^This is the lower bound for true significance

**Table 5:** Test of homogeneity of variances

Test of homogeneity of variances					
		Levene statistic	df1	df2	Sig
Displacement	Based on mean	.831	5	24	.540
	Based on median	.595	5	24	.704
	Based on median and with adjusted df	.595	5	22.408	.704
	Based on trimmed mean	.821	5	24	.547

To further compare the sacral displacement among the six groups, analysis of variance (ANOVA) was then applied and revealed a significant difference (*p* < 0.05) (Table 6).

**Table 6:** Analysis of variance (ANOVA)

Analysis of variance (ANOVA)					
Displacement	Sum of squares	df	Mean square	F	Sig
Between groups	.210	5	.042	17.192	.000
Within groups	.059	24	.002		
Total	.268	29			

A test for pairwise comparisons, i.e., the least significant difference (LSD) test, was performed on the six groups to verify the relationships between them. The LSD test confirmed that there was no significant difference between Groups 3 and 6, 1 and 5, or 2 and 4, while there was a significant difference between all other pairs of groups (Table 7).

**Table 7:** Least significant difference (LSD) test for multiple comparisons

Least significant difference **(LSD) test for multiple comparisons**						
LSD						
(I) Group	(J) Group	Mean difference (I-J)	Std. error	Sig	95% Confidence interval	
					Lower bound	Upper bound
1	2	-.1096800^a^	.0312499	.002	-.174177	-.045183
	3	.0750600^a^	.0312499	.024	.010563	.139557
	4	-.1052400^a^	.0312499	.003	-.169737	-.040743
	5	-.0109200	.0312499	.730	-.075417	.053577
	6	.1154200^a^	.0312499	.001	.050923	.179917
2	1	.1096800^a^	.0312499	.002	.045183	.174177
	3	.1847400^a^	.0312499	.000	.120243	.249237
	4	.0044400	.0312499	.888	-.060057	.068937
	5	.0987600^a^	.0312499	.004	.034263	.163257
	6	.2251000^a^	.0312499	.000	.160603	.289597
3	1	-.0750600^a^	.0312499	.024	-.139557	-.010563
	2	-.1847400^a^	.0312499	.000	-.249237	-.120243
	4	-.1803000^a^	.0312499	.000	-.244797	-.115803
	5	-.0859800^a^	.0312499	.011	-.150477	-.021483
	6	.0403600	.0312499	.209	-.024137	.104857
4	1	.1052400^a^	.0312499	.003	.040743	.169737
	2	-.0044400	.0312499	.888	-.068937	.060057
	3	.1803000^a^	.0312499	.000	.115803	.244797
	5	.0943200^a^	.0312499	.006	.029823	.158817
	6	.2206600^a^	.0312499	.000	.156163	.285157
5	1	.0109200	.0312499	.730	-.053577	.075417
	2	-.0987600^a^	.0312499	.004	-.163257	-.034263
	3	.0859800^a^	.0312499	.011	.021483	.150477
	4	-.0943200^a^	.0312499	.006	-.158817	-.029823
	6	.1263400^a^	.0312499	.000	.061843	.190837
6	1	-.1154200^a^	.0312499	.001	-.179917	-.050923
	2	-.2251000^a^	.0312499	.000	-.289597	-.160603
	3	-.0403600	.0312499	.209	-.104857	.024137
	4	-.2206600^a^	.0312499	.000	-.285157	-.156163
	5	-.1263400^a^	.0312499	.000	-.190837	-.061843

Group 1: L5NS1; Group 2: L5NS2; Group 3: L5LS1; Group 4: L5LS2; Group 5: L5NS12; Group 6: L5LS12

^a^The mean difference is significant at the 0.05 level

The best vertical stability of the sacrum among the six groups of models occurred in the L5LS12 model, followed by the L5LS1 model, with no significant difference between the two models in terms of sacral displacement. The next best stability was observed in the L5NS1 and L5NS12 models, with no significant difference in sacral displacement between these two models. The maximum vertical sacral displacement occurred in the L5LS2 and L5NS2 models, with no significant difference between them. Thus, by comparing the vertical displacement of the sacrum among the six groups, we found the following phenomena:

1. The best vertical stability of the internal fixation model was achieved when the S1 segment was fixed with lengthened sacroiliac screws, followed by when the S1 segment was fixed using normal sacroiliac screws.
2. The 2 models in which the S2 segment alone was fixed using sacroiliac screws showed the worst vertical stability of all the models. Even when the S2 segment was fixed with a lengthened sacroiliac screw alone, the vertical stability of the model was worse than that of the model when the S1 segment was fixed with a normal sacroiliac screw alone.
3. There was no significant difference in vertical stability between the S1 + S2 dual-segment fixation model and the S1-segment fixation model.

### Maximum pressure on implants

**Fig. 11:**
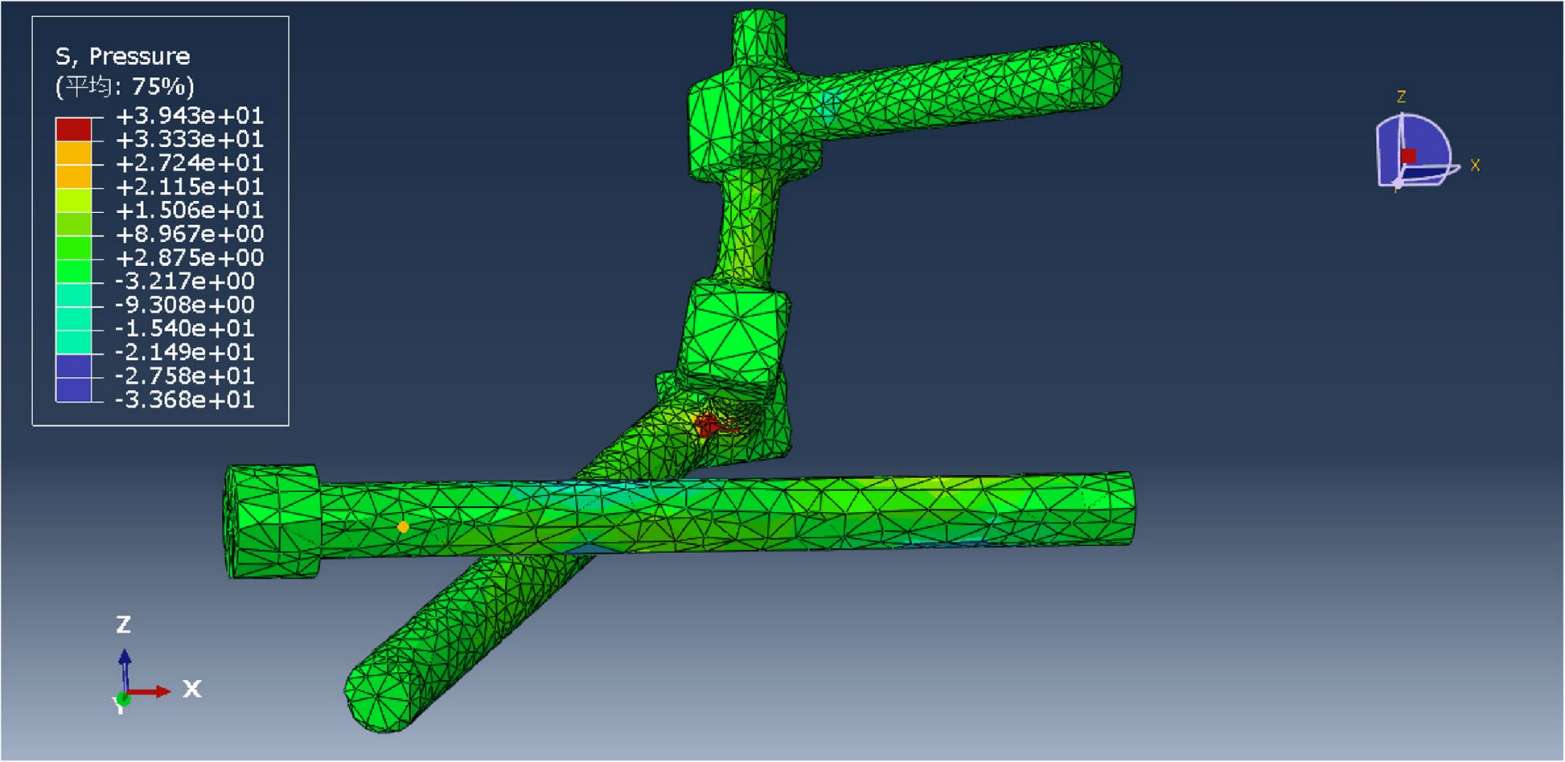
Pressure distribution of the L5NS1 model

**Fig. 12:**
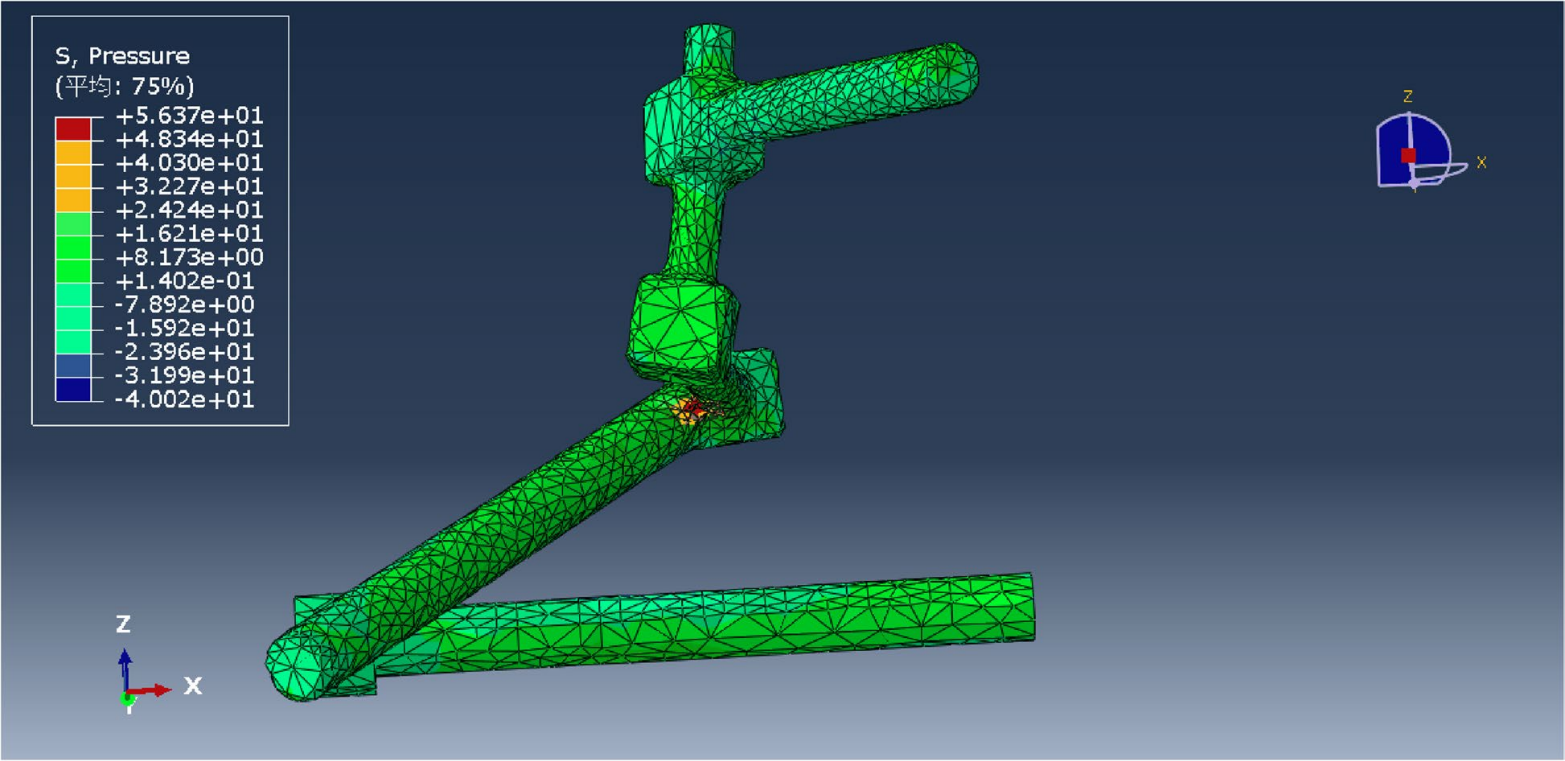
Pressure distribution of the L5NS2 model

**Fig. 13:**
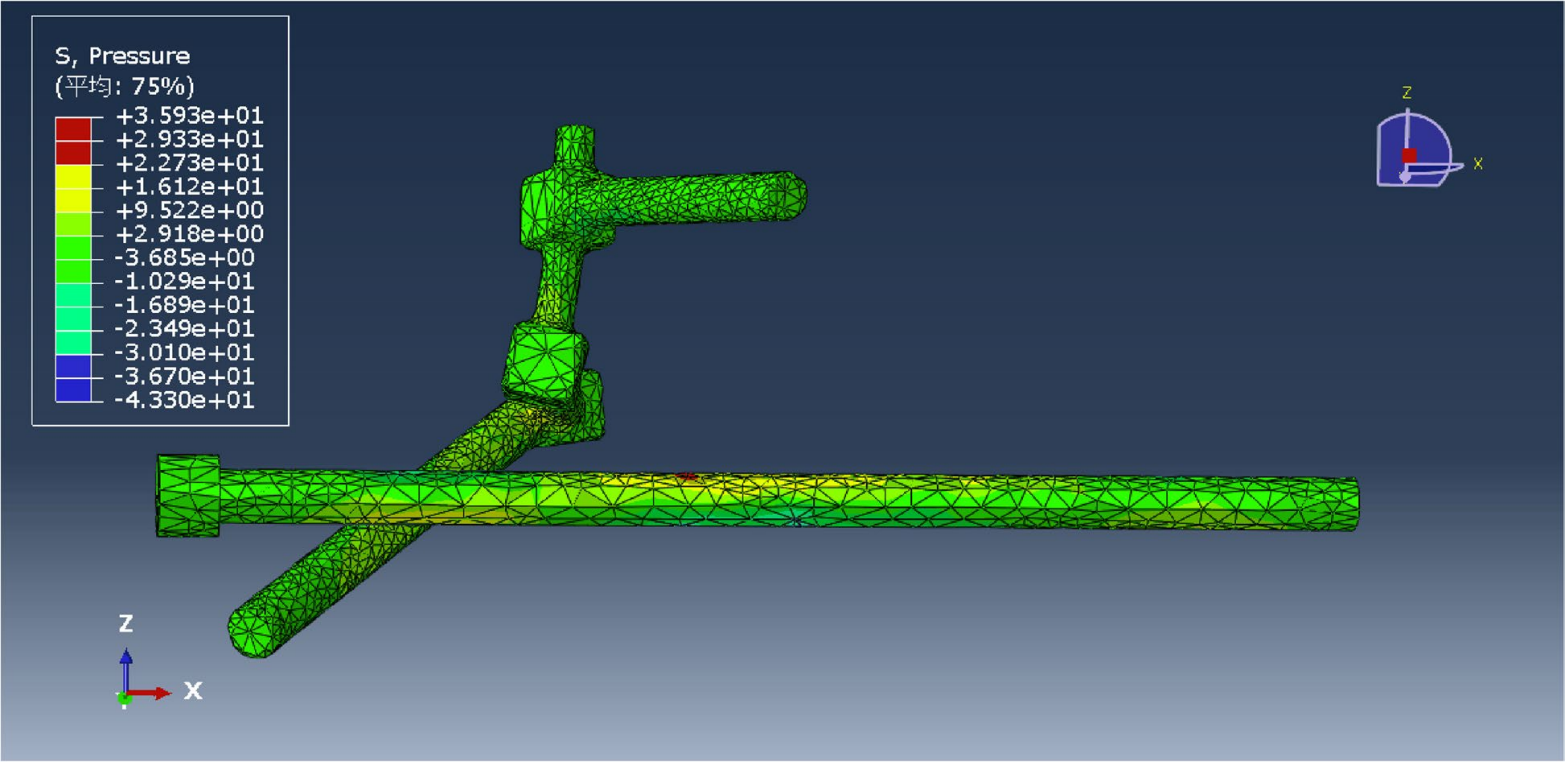
Pressure distribution of the L5LS1 model

**Fig. 14:**
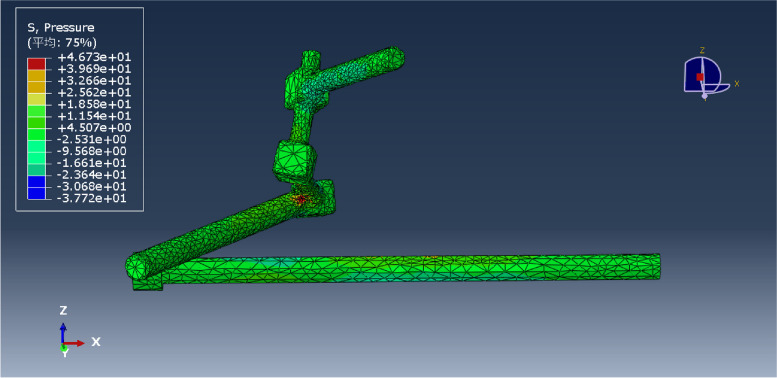
Pressure distribution of the L5LS2 model

**Fig. 15:**
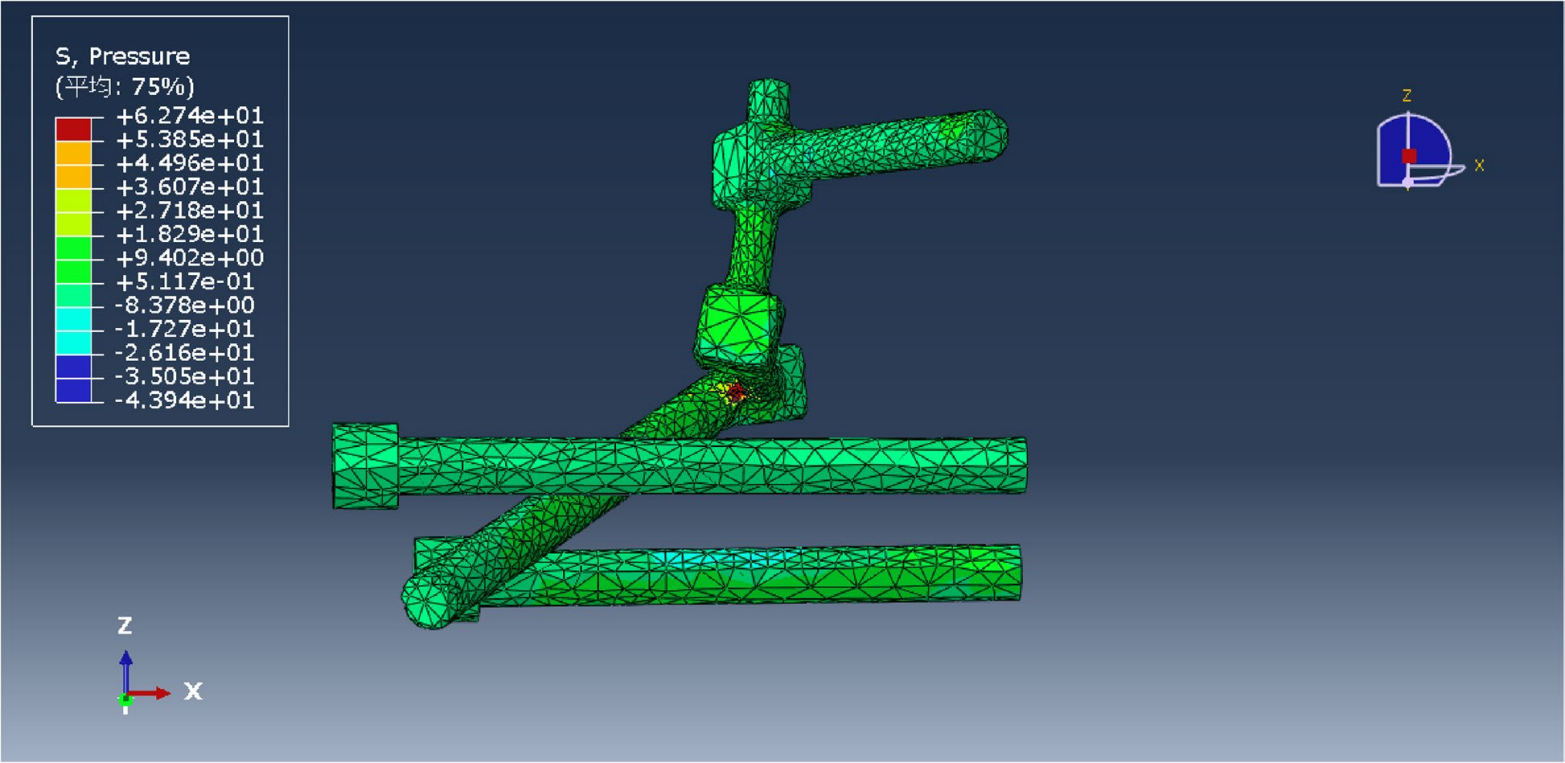
Pressure distribution of the L5NS12 model

**Fig. 16:**
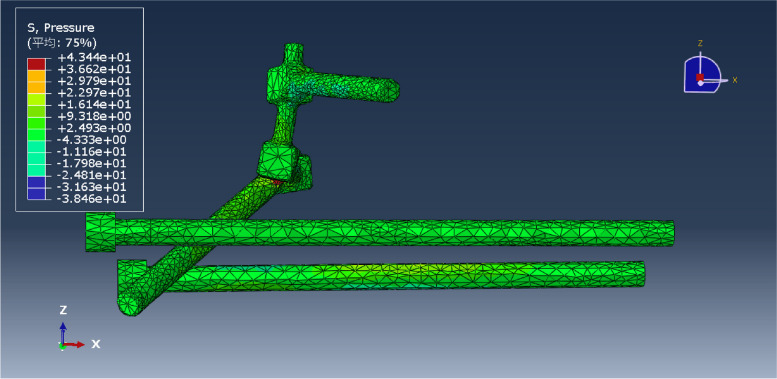
Pressure distribution of the L5LS12 model

**Fig. 17:**
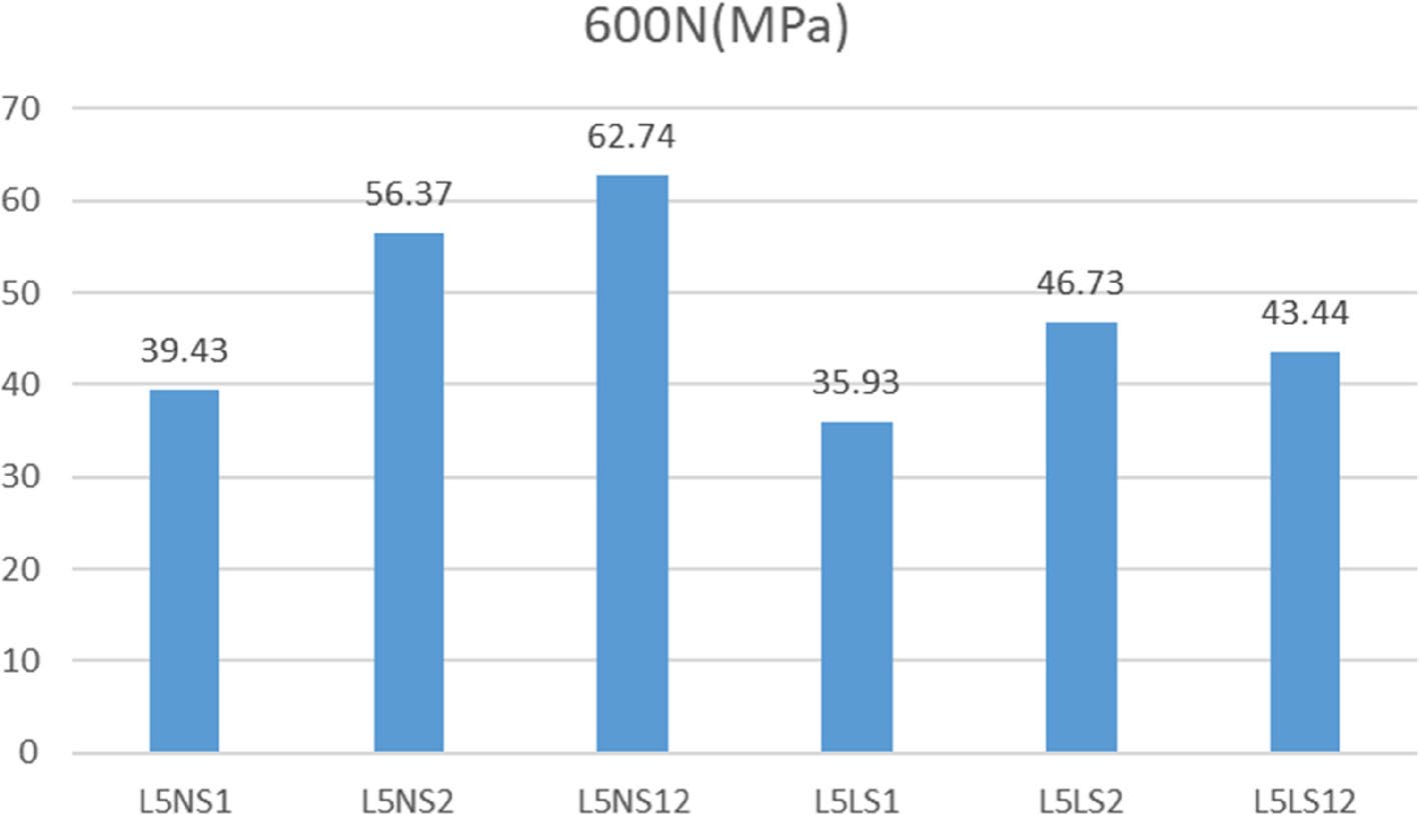
Maximum pressure on the surface of the implant in a standing position

Under a vertical force of 600 N, the pressure distribution of each model was obtained, and the maximum pressure of each was recorded (Figs. 11, 12, 13, 14, 15 and 16). Comparison of the maximum pressure under a vertical force of 600 N showed a trend of L5LS1 < L5NS1 < L5LS12 < L5LS2 < L5NS2 < L5NS12, as shown in Fig. 17. The smallest value of 35.93 MPa was recorded for the L5LS1 model, followed by 39.43 MPa for the L5NS1 model. Therefore, the pressure distribution was more uniform in the L5LS1 and L5NS1 models, and the maximum local pressure on the components was smaller in these models among all six internal fixation models. In L5NS2 and LSNS1S2, the local pressure was more concentrated, and the maximum pressure on the components was more significant. Comparing the pressure distributions of the six fixation models, we found that the maximum pressure in the L5LS1 model was on the lengthened sacroiliac screw. The maximum pressure in the other five models was on the iliac side of the lumbar fixation device attached by the iliac screw. Overall, higher pressure values were observed on the sacroiliac screws near the fracture line and at the site of iliac screw connection with the device for iliolumbar fixation (Figs. 11, 12, 13, 14, 15 and 16).

## Discussion

The advantage of iliolumbar fixation lies mainly in reestablishing spinopelvic stability in the vertical direction. Kach and Trentz [22] first applied the pedicle screw system of the spine to the ilium and referred to it as spinal-pelvic fixation or iliolumbar fixation. The disadvantages of this fixation method are obvious; first, there is limited resistance to pelvic rotation; and second, there is difficulty in the surgical repositioning of rotational pelvic deformities. Schildhaner al. [11] showed that this type of fixation only enhances vertical stability. In contrast, rotational stability cannot be accomplished by 2-point fixation in the vertical direction; thus, there is still rotational instability of the posterior pelvic ring that precludes early weight-bearing.

Sacroiliac screws are tension screws that are inserted into the sacrum through both cortices of the ilium and one side of the sacrum. There is also a lengthened sacroiliac screw that passes from the iliac bone on one side through the sacrum to the opposite iliac bone. Zhao al. [15, 16] conducted a biomechanical study on lengthened sacroiliac screws and concluded that the increased length of these screws better distributes the load in the vertical direction than that of normal sacroiliac screws, reducing stress concentrations and counteracting displacement. These longer sacroiliac screws have more threads than shorter sacroiliac screws and can be anchored in the bilateral iliac cortical bone, resulting in increased holding power. However, this method also has disadvantages, such as higher technical requirements, a higher risk of neurovascular damage, a longer operation, and more bleeding.

Triangular osteosynthesis is a method for spinopelvic fixation that involves the use of a longitudinal fixation device combined with a transverse fixation device, which can be either a plate or a sacroiliac screw. This method allows immediate full weight-bearing and functional exercise for patients with C-shaped pelvic fractures. Spinopelvic fixation can be performed with L4 or L5 pedicle screws combined with lateral sacral plates or iliac pedicle screws. Schildhauer et al. [12] compared the results of biomechanical experiments on early weight-bearing in patients with unstable sacral fractures and concluded that the stability of the posterior pelvic ring was significantly higher with triangular osteosynthesis than with sacroiliac screw fixation [23]. The use of iliolumbar fixation combined with sacroiliac screws is now a more common mode of the clinical application of triangular osteosynthesis. There are some potential complications of triangular osteosynthesis, such as nerve injury, vascular injury, and soft tissue injury. The application of orthopaedic robots and orthopaedic navigation methods has reduced the complications of triangular osteosynthesis surgery. Furthermore, some scholars have reported the application of triangular osteosynthesis in a minimally invasive manner to reduce soft tissue injury during surgery, with good results [24].

According to the literature, there have been few biomechanical studies on triangular osteosynthesis. Additionally, there have been no relevant studies on the effect of sacroiliac screw position and length on the overall mechanical characteristics of triangular osteosynthesis. Thus, we designed six fixation models for biomechanical analysis. The sacral fracture model simulated a unilateral vertical fracture of the sacrum through the sacral foramen. Unilateral L5 + iliac fixation was used to simulate unilateral iliolumbar fixation in all fixation models. The fracture in the model did not involve the L5/S1 tuberosity; thus, the L5 vertebral body was intact, and fixation of the L5 segment alone was more appropriate. We applied sacroiliac screws at S1 and S2 alone and S1 + S2 together to compare the effect of different fixation configurations on sacral stability. We also applied normal and lengthened sacroiliac screws separately to compare the effect of sacroiliac screw length on the stability of sacral fixation.

After fixation of the sacral fracture, the stability of the sacrum can be represented by the displacement of the upper surface of the sacrum. Of the six fixation models, the L5LS12 and L5LS1 models showed the least vertical displacement of the sacrum. The difference in vertical displacement between the two models was not statistically significant, indicating that these two models provided better vertical stability than the other models in this study. However, adding a lengthened sacral screw at S2 did not further increase stability; Although intraoperative imaging devices(O-arm) can significantly improve the safety of sacroiliac screws [25], adding a single lengthened low skeletal screw would increase the risk of neurovascular injury and prolong the operation but not provide better vertical sacral stability. Therefore, for unilateral longitudinal sacral fractures (AO C1.3), we prefer S1-segment lengthened sacroiliac screw fixation to obtain satisfactory stability.

However, not all patients had S1 lengthened screw safety access, but S2lengthened screw safety access was present in most patients. In patients lacking S1 lengthened screw safe passage. S1 segmental Normal Sacroiliac (L5NS1) screw fixation was superior to S2 segmental lengthened sacroiliac screws (L5LS2). The S1 Normal Sacroiliac screws were technically easier to perform and had fewer complications. Our study also demonstrated that the stability of fracture fixation with the S1 normal sacroiliac screw was superior to that with the S2 normal sacroiliac screw. There was no statistically significant difference in stability between S1 + S2 segmental normal sacroiliac screw fixation and S1 segmental normal sacroiliac screw fixation.

In addition, in the case that the S1 screw tract cannot be used and that an S2 sacroiliac screw must be used, there will be no significant difference in vertical sacral stability between the lengthened and normal S2 sacroiliac screws. In such cases, given that a lengthened S2 screw is more difficult to implant, a normal S2 sacroiliac screw can be used.

In this study, the maximum pressure on internal fixation components was analysed and used to assess the risk of implant fracture. Typically, von Mises stress is used in the theoretical assessment of the risk of internal fixation instrumentation fracture. Considering the actual rehabilitation of patients, internal fixation screw fracture is often the result of metal fatigue under chronic repetitive stress. The local pressure on internal fixation components reflects, to a certain extent, the local stress on these components and can serve as an indicator of the risk of fatigue-induced fracture of internal fixation implants in a manner that is more in line with the patient recovery process. In the present study, the L5LS1 and L5NS1 models showed a more uniform pressure distribution and a lower maximum local pressure on the internal fixation components, which resulted in a lower risk of fatigue-induced fracture in these two fixation modes. In the L5NS2 and LSNS1S2 models, the local pressure was more concentrated, and the maximum local pressure on the implants was higher, resulting in a higher risk of fatigue-induced fracture in these two models.

In our study, we found that additional S2 sacroiliac screw fixation did not significantly enhance the vertical stability of the sacrum and did not significantly reduce the compression force on the endplate. This phenomenon was more pronounced with normal screws than with lengthened screws and may be related to the lower density of the sacral bone in the S2 segment than in the S1 segment. A previous study using Hounsfield units to assess bone density noted that the bone density of the sacral body was lower in S2 than in S1. Based on this finding, the authors cautioned against using sacroiliac screw fixation at the S2 level, as the relatively low bone density of the sacral body could lead to screw loosening and fixation failure [26].

Regarding the results of this study, the following points must be considered. First, whereas anterior ring instability is a hallmark of C-shaped pelvic ring injuries, numerous treatment strategies for anterior ring fractures may have an impact on the stability of the posterior pelvic ring. Throughout the current study, rather than simulating injury and fixation of the anterior pelvic ring, it was maintained in its normal state.Various fixation methods exist for anterior ring injuries, such as plates, hollow screws, INFIX, and external fixation frames. The biomechanical performance of different fixation methods varies. Modeling an anterior ring injury and fixation increases the workload considerably and impacts posterior ring stability compared to pride. This model's absence of anterior ring injury can be interpreted as a complete restoration of anterior ring stability using an ideal fixation method. This design excludes the influence of the front ring fixation method on the rear ring stability results. It may interfere with the post-loop stability results to some extent but does not affect the stability comparison between different fixation methods for triangular fixation. Second, we kept numerous significant pelvic ligaments in our analysis to better mimic the stability of the pelvis. Additionally, we did not simulate muscles to avoid any unanticipated forces that could alter the measurements. Although joint flexibility settings, muscle parameters, and other joint-related factors had a qualitative impact on the outcomes, the computations were too complex to complete the corresponding quantitative experiments.Third, The biomechanical finite element analysis did not involve a model of osteoporosis and Fragility fractures of the pelvis (FFP).

## Conclusions

When applying triangular osteosynthesis through unilateral iliolumbar fixation + sacroiliac screw fixation for unilateral vertical sacral fractures (AO C1.3, Denis II), it is recommended that a single lengthened sacroiliac screw be placed in the S1 segment to achieve the best vertical sacral stability with less compression on the internal fixation components. If it is not possible to apply a lengthened sacroiliac screw, the use of a normal sacroiliac screw in the S1 segment is recommended. The additional use of an S2 screw does not significantly increase the vertical stability of the sacrum.

## Data Availability

The datasets used and analysed during the current study are available from the corresponding author on reasonable request.
